# Interaction Between Neurogenic Stimuli and the Gene Network Controlling the Activation of Stem Cells of the Adult Neurogenic Niches, in Physiological and Pathological Conditions

**DOI:** 10.3389/fcell.2020.00211

**Published:** 2020-04-07

**Authors:** Manuela Ceccarelli, Giorgio D’Andrea, Laura Micheli, Felice Tirone

**Affiliations:** Institute of Biochemistry and Cell Biology, National Research Council (IBBC-CNR), Rome, Italy

**Keywords:** adult neurogenesis, neural stem cells, self-renewal, stem cell quiescence/activation, neurogenic stimuli, gene network, aging, depression

## Abstract

In the adult mammalian brain new neurons are continuously generated throughout life in two niches, the dentate gyrus of the hippocampus and the subventricular zone. This process, called adult neurogenesis, starts from stem cells, which are activated and enter the cell cycle. The proliferative capability of stem cells progressively decreases during aging. The population of stem cells is generally quiescent, and it is not clear whether the potential for stem cells to expand is limited, or whether they can expand and then return to quiescence, remaining available for further activation. Certain conditions may deregulate stem cells quiescence and self-renewal. In fact we discuss the possibility of activation of stem cells by neurogenic stimuli as a function of the intensity of the stimulus (i.e., whether this is physiological or pathological), and of the deregulation of the system (i.e., whether the model is aged or carrying genetic mutations in the gene network controlling quiescence). It appears that when the system is aged and/or carrying mutations of quiescence-maintaining genes, preservation of the quiescent state of stem cells is more critical and stem cells can be activated by a neurogenic stimulus which is ineffective in normal conditions. Moreover, when a neurogenic stimulus is in itself a cause of brain damage (e.g., kainic acid treatment) the activation of stem cells occurs bypassing any inhibitory control. Plausibly, with strong neurogenic stimuli, such as kainic acid injected into the dentate gyrus, the self-renewal capacity of stem cells may undergo rapid exhaustion. However, the self-renewal capability of stem cells persists when normal stimuli are elicited in the presence of a mutation of one of the quiescence-maintaining genes, such as p16Ink4a, p21Cip1 or Btg1. In this case, stem cells become promptly activated by a neurogenic stimulus even during aging. This indicates that stem cells retain a high proliferative capability and plasticity, and suggests that stem cells are protected against the response to stimulus and are resilient to exhaustion. It will be interesting to assess at which functional degree of deregulation of the quiescence-maintaining system, stem cells will remain responsive to repeated neurogenic stimuli without undergoing exhaustion of their pool.

## Adult Neurogenesis in the Dentate Gyrus and Subventricular Zone (SVZ) and Self-Renewal of Stem Cells

Neurogenesis persists in the mammalian brain in two specific neurogenic niches, the subgranular zone (SGZ) of the hippocampal dentate gyrus and the subventricular zone (SVZ) adjacent to lateral ventricles, where new neurons are generated throughout life from stem cells (Kempermann et al., 2015; Lim and Alvarez-Buylla, 2016).

Hippocampal adult neurogenesis is necessary for learning and memory, as it contributes to enhance the ability to distinguish between similar memory patterns; this ability, defined pattern separation, is in-built in the dentate gyrus circuitry, but is greatly improved by the addition of new neurons to the existent circuits (Farioli-Vecchioli et al., 2008; Aimone et al., 2011; Sahay et al., 2011b; Tirone et al., 2013). As for the SVZ, the new neurons generated from stem cells during adulthood migrate to the olfactory bulb, where they contribute to the olfactory function (Lim and Alvarez-Buylla, 2016). Remarkably, these SVZ neurons can contribute to repair brain areas, damaged for instance by stroke or trauma (Christie and Turnley, 2013).

Neural stem cells are mainly quiescent in both neurogenic niches (Urbán et al., 2019). In the SGZ, adult neurogenesis involves the activation of stem cells with radial glia-like morphology, labeled by GFAP, Sox2, nestin, and named type-1 cells, as proposed by Kempermann et al. (2004) (Komitova and Eriksson, 2004; Steiner et al., 2006). Neural stem cells divide mostly asymmetrically (Kempermann et al., 2004; Bonaguidi et al., 2011; Encinas et al., 2011), giving rise to rapidly proliferating progenitor cells (type-2 and type-3; Filippov et al., 2003; Fukuda et al., 2003; Kronenberg et al., 2003; Steiner et al., 2004), which mature into post-mitotic neurons (stage 5) and then into terminally differentiated neurons (stage 6; Brandt et al., 2003).

Also in the SVZ, the other neurogenic niche, radial glia-like stem cells expressing GFAP (B cells) produce proliferating transient amplifying cells, which then mature into neuroblasts (DCX-positive, A cells) that finally migrate to the olfactory bulb (Lim and Alvarez-Buylla, 2016).

Considering that a network of genes controls the quiescence of stem cells, we will discuss how neurogenic stimuli and the quiescence-maintaining gene network interact, i.e., whether a neurogenic stimulus can activate stem cells in physiological conditions and how the neurogenic stimulus response is modulated by quiescence-maintaining genes. This should help define the extent by which the pool of stem cells is resilient to depletion.

There are two theories about the process of self-renewal of the stem cell pool in the SGZ, one proposing a repeated self-renewal of stem cell, and another proposing a “disposable stem cell” model. In the first model, a quiescent stem cell after activation in physiological conditions may undergo several rounds of asymmetrical division generating a progeny that differentiates into neuron or astroglia; but stem cells may also expand dividing symmetrically, and in either division mode they can return to a quiescent state, remaining available for further activation (Bonaguidi et al., 2011). In the “disposable stem cell” model, after activation, the stem cell divides only asymmetrically a number of times and then conclusively differentiates into astrocyte or neuron, thus depleting the pool (Encinas et al., 2011). A further insight in this latter model shows that when the activation is elicited by a stimulus of medium or strong intensity (kainic acid), which mimics epileptiform activity, then the division mode shifts from asymmetric to symmetric with prevalent astrocytogenesis and accelerated depletion of the pool (Sierra et al., 2015).

Certainly, it is not possible to exclude that differences in the mouse models used by Bonaguidi et al. (2011) and Encinas et al. (2011) may account for part of the differences observed. However, other studies have shown that not all neural stem cells are rapidly depleted and that part of them returns to quiescence in both the SGZ and the SVZ, being possibly responsible for the preservation of the stem cell pool and neurogenesis in old age (Urbán et al., 2016; Obernier et al., 2018; Pilz et al., 2018). In fact, Pilz et al. (2018) propose a model in which radial glia stem cells can enter sporadically into the cell cycle, with the possibility of shuttling back and forth between quiescence and activity. Moreover, the population analyzed by Pilz et al. (2018) was limited to the Ascl1-positive cells, which leaves the possibility of other neural stem cell populations remaining longer in quiescence. Furthermore, Urbán et al. (2016) show that indeed stem cells of the dentate gyrus can reenter quiescence after having been activated, provided the E3-ubiquitin ligase Huwe1 is degraded, which ultimately inhibits the increase of cyclin D1. The primed stem cells reentering quiescence are in a resting state not as deep as the original quiescent state but they can nonetheless sustain the stem cell pool, since the authors show that if stem cells fail to return to quiescence, then the proliferative stem cell pool is depleted (Urbán et al., 2016).

Likewise, a model has also been proposed for SVZ stem cells, whereby about one third of them self-renew symmetrically, thus preserving the pool and remaining available for further activation; the remaining two thirds of stem cells divide generating progeny with an expansion stronger than in the SGZ, thus consuming the pool (Calzolari et al., 2015; Obernier et al., 2018). This model keeps open the possibility of expansion also during aging, when, however, neurogenesis is reduced. A further comprehensive model for long-term self renewal of SVZ stem cells has been proposed by Basak et al. (2018), where stem cells reversibly return to quiescence after proliferation depending on the state of the niche, either proliferative or quiescent. Interestingly, quiescent SVZ cells, once primed, can reenter the cell cycle after injury, i.e., either 5-fluorouracil (Basak et al., 2018) or ischemia (Llorens-Bobadilla et al., 2015), compensating the depletion of neural stem cells and thus revealing a latent plasticity.

Thus, besides the actual model of stem cell self-renewal in the SGZ or SVZ, it seems that at least in physiological conditions, self-renewal can be reversible from quiescence to activity and vice versa, thus opening possibilities of a multi-faceted process that can be activated upon demand or stimulus.

Stem cell heterogeneity is another factor that comes into play. Indeed, stem cells of the dentate gyrus have been shown to display different degrees of functionality, some of them being neurogenic and other multipotent and more able to self-renew (DeCarolis et al., 2013; Ibrayeva et al., 2019 [Preprint]). However, this points to a functional heterogeneity and to differences between transgenic mouse models rather than to the existence of specific subpopulations. This possibility is suggested also by RNA sequencing analyses of single cells, which indicate that discrete subpopulations of neural stem cells in the dentate gyrus cannot be identified, being highly heterogeneous, with a continuum of cells that progressively downregulate genes involved in the maintenance of quiescence, such as cell cycle genes (Artegiani et al., 2017). In the SVZ as well it has been observed that neural stem cells from different domains, correlated to the expression of specific transcription factors (Nkx.2, Zic, Gli1), originate different interneurons within the anterior ventral SVZ (Merkle et al., 2014).

The presence in the dentate gyrus of heterogeneous populations of stem/progenitor cells may be suitable to respond to different tissue requests (Lugert et al., 2010). Moreover, these populations may be regulated in different ways and may thus respond selectively to environmental cues (Bonaguidi et al., 2012).

## Control of Stem Cells Activation in the Dentate Gyrus and SVZ by Genes/Factors

The quiescence, proliferation, and differentiation of stem cells is coordinated by a complex balance of environmental cues (Fuentealba et al., 2012). These include pro-proliferative factors/stimuli such as VEGF (Licht et al., 2016) and Noggin (Bonaguidi et al., 2008), or factors involved in maintaining the quiescence, such as BMPs (Mira et al., 2010), Id4 (Blomfield et al., 2019), FoxO3 (Paik et al., 2009), Notch (Ables et al., 2010; Ehm et al., 2010; Lugert et al., 2010), p21Cip1 (Porlan et al., 2013), Btg1 (Farioli-Vecchioli et al., 2012), p27Kip1 (Andreu et al., 2015), or favoring differentiation such as GABA (Tozuka et al., 2005), NeuroD1 (Richetin et al., 2015), NeuroD2 (Micheli et al., 2017), and Tis21/Btg2 (Farioli-Vecchioli et al., 2008). While BMPs maintain quiescence of stem cells in the dentate gyrus, conserving them in an undifferentiated state, overexpression of the antagonist Noggin recruits quiescent stem cells to the cycle but is followed by decreased stem cell division and partial depletion of non-radial cells, i.e., of progenitor cells, and of neurons (Mira et al., 2010). Similarly, Btg1 maintains the quiescence of stem cells in the dentate gyrus and SVZ, since its deletion induces a proliferative burst of stem and progenitor cells early after birth, followed by a decline of proliferation and apparent depletion of the stem cells and of mature neuron generation in the dentate gyrus and SVZ (Farioli-Vecchioli et al., 2012, 2014; Micheli et al., 2018a). Also the Notch pathway maintains the quiescence of stem cells, as its inactivation induces, within 2 months, a decrease of the number of stem cells, accompanied by increased generation of neurons and consequent depletion of the stem cell pool (Ables et al., 2010; Ehm et al., 2010). The mechanism by which Notch acts is by activating its effector Hes1, which in turn suppresses the expression of Ascl1, activator of stem cells exit from quiescence (Sueda et al., 2019). p27Kip1 has been demonstrated to maintain dentate gyrus stem cells in quiescence, as its deletion leads to great increase of proliferating radial stem cells, progenitor cells and neurons; it is not clear, however, whether this leads to a decreased production of stem/progenitor cells in the long-term (Andreu et al., 2015). Id4, instead, maintains SGZ stem cells in quiescence by turning off Ascl1 – sequestering the Ascl1 heterodimerization partner E47 – thus preventing the entry into the cell cycle (Blomfield et al., 2019). Also FoxO3 maintains SGZ and SVZ stem cells quiescent with a mechanism that may involve competition with Ascl1 for DNA binding, but also downregulation of metabolic genes (Paik et al., 2009; Renault et al., 2009).

## Neurogenic Stimuli Activating Stem Cells in Neurogenic Niches of Adult, Aged and Brain Pathology Models

A powerful control of stem cell quiescence is also revealed by exogenous neurogenic stimuli, such as physical exercise (running), antidepressants, or nutrient molecules or by learning itself. Complex cellular processes are necessary to translate these stimuli into neurogenic changes.

### Physical Exercise in the Dentate Gyrus and SVZ

It is known that running acts on neurogenesis, neural circuitry, neurotransmission, neurotrophins, vasculature, and synaptic plasticity. Moreover, peripheral organs, such as muscle, liver and adipose tissue are influenced by running and release specific systemic factors that stimulate neurotrophins and neurogenesis in the hippocampus (Vivar et al., 2013).

Several reports indicate that physical exercise (running) stimulates adult neurogenesis in the dentate gyrus by inducing the proliferation of progenitor cells (type-2 and type-3) without, however, being able to activate type-1 stem cells (Kronenberg et al., 2003; Steiner et al., 2008; Brandt et al., 2010; Farioli-Vecchioli et al., 2014). There is also evidence that running increases BrdU^+^Sox2^+^ cells (Suh et al., 2007), which, however, comprise not only stem cells but also proliferating type-2ab transit amplifying cells (Komitova and Eriksson, 2004; Steiner et al., 2006). Moreover, according to Lugert et al. (2010), a subpopulation of quiescent radial neural stem cells, expressing Hes5, respond to running (see Table 1). A very interesting report indicates that the transition of stem cells from the quiescent to proliferative state following running corresponds to a conversion from high to lower oxidative state and to a decrease of expression of quiescence-maintaining genes, such as Btg1, Btg2, p21Cip1 (Adusumilli et al., 2019 [Preprint]). Interestingly, the activation of neural stem cells by metabolic mechanisms appears to be a general concept and not exclusively related to the stimulus of running. Indeed, the mechanisms of the shift from quiescence to activity for hippocampal stem cells is also dependent on the metabolism of fatty acids, since the inhibition of their breakdown (i.e., of their oxidation) leads to quiescence, while the activation of oxidation triggers the proliferation of stem cells with cell cycle re-entry, even after BMP4-mediated induction of quiescence (Knobloch et al., 2017). Likewise, transition from quiescence to activation of stem cells has been found to be associated with downregulation of glycolytic metabolism, of Notch and BMP signaling (Llorens-Bobadilla et al., 2015).

**TABLE 1:** Proliferative activation of stem and progenitor cells of the adult and aged dentate gyrus by different neurogenic stimuli.

System (dentate gyrus)	Neurogenic stimulus	Activation of stem cells (type-1; BrdU^+^ GFAP^+^ nestin^+^ Sox2^+^)	Activation of progenitor cells (type-2-3)	Cognitive effects	References
Adult	Running	NO	YES	Rescue of contextual memory	Suh et al., 2007; Farioli-Vecchioli et al., 2014; Kronenberg et al., 2003; Steiner et al., 2008; Brandt et al., 2010;
	Running	YES (Hes5^+^)	–	–	Lugert et al., 2010
	Fluoxetine	NO	YES (and increased neuron survival)	–	Encinas et al., 2006; Micheli et al., 2017, 2018a;
	Fluoxetine (after global ischemia)	YES	YES		Couillard-Despres et al., 2009; Khodanovich et al., 2018
	Learning (enriched environment)	NO	NO (but increased survival of neurons)	Improved spatial learning	Kronenberg et al., 2003; Kempermann et al., 1997;
	Learning (enriched environment preceded or not by hypoxia)	YES	YES (and increased number of neurons)	Improved spatial learning	Salmaso et al., 2012
	Learning (spatial learning)	–	YES (BrdU^+^) (but increased survival of neurons)	–	Gould et al., 1999; Ambrogini et al., 2000; Döbrössy et al., 2003; Epp et al., 2007
	Nutrient: Hydroxytyrosol	NO*	NO (but increased survival of new neurons)	–	D’Andrea et al., 2020
	Nutrient: Luteolin (in mouse model of Down syndrome and Alzheimer disease Ts65Dn)	YES***	YES	Improved spatial learning and object recognition	Zhou et al., 2019
	Nutrient: Caffeoylquinic acid (in senescence-accelerated prone 8 mouse [SAMP8])	YES	– (increased generation of new neurons BrdU^+^NeuN^+^)	Improved spatial memory	Sasaki et al., 2019
	Nutrient: Astragaloside VI (after ischemia)	YES	–	Rescue of spatial memory	Chen et al., 2019
	Nutrient: n-3 PUFA	–	YES	Improved spatial memory	Cutuli et al., 2014
	Electromagnetic fields	YES	YES	Improved spatial learning	Leone et al., 2014
Adult (after corticosterone-induced depression-like state)	Running	–	YES	Rescue of spatial memory	Yau et al., 2012
Aged	Nutrient: Hydroxytyrosol	YES**	YES (and increased survival of neurons)	–	D’Andrea et al., 2020
	Running	NO	YES	Rescue of place recognition memory	Siette et al., 2013; Micheli et al., 2019
	Fluoxetine	NO	NO (and no increase of neuron survival)	No rescue of visuospatial deficit; enhancement of contextual memory and spine density	Micheli et al., 2018a; Couillard-Despres et al., 2009; Li et al., 2015; McAvoy et al., 2015
	Learning (enriched environment)	–	NO (increased survival of neurons)	No improved spatial learning	Kempermann et al., 1998

*Ki67^+^Sox2^+^GFAP^+^ **Ki67^+^Sox2^+^ (Type-1-2a) ***Nestin^+^ (Type-1-2ab) or GFAP^+^.

Therefore, the metabolic activation of fatty acid and of glycolysis play roles in the process of stem cells activation, thus suggesting common mechanisms of activation of neurogenesis by running (for instance, through adiponectin, Yau et al., 2014) and nutrients (see also section “Nutrients as Activators of Stem Cells in Adult and Aged Dentate Gyrus and SVZ”).

There is no ready answer as to why running (or other neurogenic stimuli) activate progenitor cells but not stem cells. We can however point out that hippocampal neurogenesis is regulated also by neural circuitry. In particular, an important role in determining the balance between quiescent or proliferative state of radial glia-like cells is played by GABAergic signals. Tonic activation of the gamma 2 receptor by GABA, released from parvalbumin-expressing interneurons, maintains quiescence of stem cells. Depletion of the receptor, conversely, induces proliferation of type-2 progenitor cells (Song et al., 2012). Interestingly, during voluntary exercise, the negative regulation of GABA_*A*_ receptors by DBI (diazepam binding inhibitor) promotes the expansion of the progenitor cell pool (Sox2^+^ and DCX^+^, i.e., type-1-2a and type-2b-3, respectively; Dumitru et al., 2017). The GABA switch may thus be a control after running of the balance existing between stem and progenitor cells.

Moreover, it is important to note that the exercise-induced activation of progenitor cells depends also on serotonin signaling, since the depletion of serotonin through tryptophan hydroxylase 2 (Tph2) knockout impairs the induction of proliferation of progenitor cells by exercise. Surprisingly, Tph2 knockout displays a decrease of Sox2^+^GFAP^+^ (type-1) cells, which resumes to normal level after running, probably as a consequence of an adaptation aimed at maintaining homeostasis of the neurogenic niche (Klempin et al., 2013). Another report indicated that the 5-HT3 receptor is specifically required for the exercise-induced SGZ neurogenesis (of progenitor cells, BrdU^+^DCX^+^) and antidepressant effect, while it is not required for exercise-induced learning (Kondo et al., 2015).

Thus, the neurogenic stimulation of progenitor cells in SGZ by exercise, is tightly controlled by neural circuits, in addition to the network of cell cycle genes such as p16Ink4a and Btg1 (see below section “Interaction Between Genes Controlling Stem Cell Activation in the Dentate Gyrus or SVZ and Neurogenic Stimuli”) and of several non-cell-autonomous factors. It is worth noting that the proliferative action of running is associated with a shortening of the S-phase of dentate gyrus progenitor cells. After deletion of the cell cycle inhibitor Btg1 also stem cells undergo cell cycle shortening (Farioli-Vecchioli et al., 2014). We proposed that the acceleration of the cell cycle may stabilize the expansion of the neural progenitor cells to this stimulus (Farioli-Vecchioli and Tirone, 2015). Instead, no change of cell cycle length was observed after the neurogenic stimulus of fluoxetine (Micheli et al., 2017).

Physical exercise is unable to activate stem cells also in the dentate gyrus of aged mice as well, where, however, it is able to partially rescue the age-dependent decline of hippocampal neurogenesis and of spatial memory (Morris water maze and place recognition tests; van Praag et al., 2005; Marlatt et al., 2012; Siette et al., 2013; Micheli et al., 2019). Moreover, running is able to activate progenitor cells and rescue a spatial memory deficit also in conditions of reduced hippocampal neurogenesis in a depression-like state induced by corticosterone treatment, but it is not defined whether also stem cells are reactivated in these conditions (Yau et al., 2012; Table 1).

In the SVZ of adult mice, running is not effective as an activator of stem cells (B cells; Brown et al., 2003; Mastrorilli et al., 2017), although prolonged running activates the proliferation of neuroblasts (A cells; Bednarczyk et al., 2009). In aged mice, however, running activates SVZ stem cells (neurospheres) as well as neuroblasts (Blackmore et al., 2009; Table 2).

**TABLE 2:** Activation of stem cells by neurogenic stimuli in the adult, aged and pathological SVZ.

System (SVZ)	Neurogenic stimulus	Activation of SVZ stem cells (Ki67/BrdU^+^GFAP or nestin/Sox2^+^)	Activation of SVZ neuroblasts/neurons (Ki67/Brdu^+^ and/or DCX^+^ or NeuN^+^)	Cognitive effects	References
Adult	Running	NO	YES (DCX^+^ or BrdU/Ki67^+^) *(prolonged)*	–	Bednarczyk et al., 2009; Nicolis di Robilant et al., 2019
	Swimming	–	YES (BrdU^+^ and DCX^+^)	–	Chae et al., 2014
	Fluoxetine	NO	NO	–	Ohira and Miyakawa, 2011; Nasrallah et al., 2010; Kodama et al., 2004
	Nutrient: Astragaloside VI (after ischemia)	YES	–	–	Chen et al., 2019
Adult (after corticosterone-induced depression-like state)	Fluoxetine	–	YES	Rescue of depression-like state and of olfactory acuity	Siopi et al., 2016
Adult (mild stress-depressed mice)	Treadmill	–	YES	Improves olfactory discrimination	Tian et al., 2020
Adult (depressed by forced swim)	Fluoxetine	YES (neurospheres)	–	–	Hitoshi et al., 2007
Aged	Running	YES (neurospheres)	YES (Brdu^+^ cells)	–	Blackmore et al., 2009

### Antidepressant Fluoxetine in the Dentate Gyrus and SVZ

An inverse correlation between adult neurogenesis and depression or also stress – which is a powerful inducer of depression – has been found. In fact, antidepressants, and among them fluoxetine, have been demonstrated to be able to activate cell proliferation and survival of newborn neurons in the hippocampus of rats and mice, and to rescue the defect of neurogenesis induced by depression or by stress [Malberg et al., 2000; Santarelli et al., 2003; Encinas et al., 2006 (for reviews Lucassen et al., 2015; Micheli et al., 2018b; Planchez et al., 2020)]. Fluoxetine, which belongs to the class of selective serotonin reuptake inhibitors (SSRI), has in fact been thoroughly studied as neurogenic stimulus in SGZ, as in adult dentate gyrus strongly stimulates the proliferation of progenitor cells but is unable to stimulate stem cells; in aged mice, however, it is ineffective in both stem and progenitor cells, but enhances the contextual memory and the density of dendritic spines (Encinas et al., 2006; Couillard-Despres et al., 2009; Li et al., 2015; McAvoy et al., 2015; Micheli et al., 2017, 2018a). Interestingly, in a mouse model of global ischemia, fluoxetine is able to completely rescue the decrease of stem and progenitor cells (Khodanovich et al., 2018; see below a mechanism of activation of stem cells by focal ischemia in SVZ described by Llorens-Bobadilla et al., 2015) (Table 1).

In adult SVZ fluoxetine is ineffective as an activator of stem or progenitor cells (Kodama et al., 2004; Nasrallah et al., 2010; Ohira and Miyakawa, 2011); when, however, mice are subjected to a depression-like protocol by forced swim or by corticosterone treatment, then stem cells or progenitor cells, respectively, are activated by fluoxetine, with rescue of depression-like state and of olfactory acuity (Hitoshi et al., 2007; Siopi et al., 2016; Table 2).

Also in view of the antidepressant effect of fluoxetine, intensive research has been performed to investigate the effect of the serotonin pathway on progenitor cell proliferation. 5-HT1A receptors have been shown to be responsible for the activation of progenitor cells in the dentate gyrus by fluoxetine, and also for its antidepressant behavioral effect (Santarelli et al., 2003). It appears that 5-HT1A receptors are mainly involved in the self-renewal of progenitor cells rather than of stem cells, while 5-HT2 affect both their proliferation and differentiation, thus impacting also on neuron survival (Klempin et al., 2010). Paradoxically, the same increase of progenitor cell proliferation is obtained when 5-HT neurons are deleted (reviewed by Song et al., 2017), implicitly indicating that the different 5-HT receptors (about 15 subtypes) have multiple effects and that neurogenic stimuli activating 5-HT1 receptors (running, fluoxetine, enriched environment) are selectively affecting progenitor cells. Moreover, fluoxetine has been demonstrated to downregulate quite selectively the expression of the cell cycle inhibitor p21Cip1, being ineffective on p18Ink4c or p27Kip1 (Pechnick et al., 2011). However, despite the observation that the deletion of p21Cip1 induces the proliferation of stem cells in both the dentate gyrus and the SVZ (Pechnick et al., 2011; Porlan et al., 2013), fluoxetine does not activate stem cells. This may be due either to insufficient downregulation of p21Cip1 by fluoxetine or to the involvement of other quiescence-maintaining signals, such as BMPs. However, the deletion of the cell cycle inhibitor Btg1 enables fluoxetine to activate stem cells in adult and aged dentate gyrus, thus suggesting that the cell cycle control is an end-point control of quiescence pathways (Micheli et al., 2018a; see below section “Interaction Between Genes Controlling Stem Cell Activation in the Dentate Gyrus or SVZ and Neurogenic Stimuli”).

### Learning and Enriched Environment as Regulators of Stem Cells in Adult and Aged Dentate Gyrus

Concerning learning or an enriched environment as neurogenic stimuli, there is evidence indicating that they activate neurogenesis in the SGZ (Kempermann et al., 1998; Epp et al., 2007; Thuret et al., 2009; see for review Epp et al., 2013). It has been shown that an enriched environment does not affect the proliferation of stem cells nor progenitor cells, but does increase survival of new neurons generated during learning (Kempermann et al., 1997, 1998; Nilsson et al., 1999; Kronenberg et al., 2003).

Summarizing and commenting the literature about the effect of enriched environment on neurogenesis is difficult due to different experimental conditions, duration of the treatment, and age and gender employed. There are mechanisms in common between environmental enrichment and running, such as activation of neural circuits whose neurotransmitters are acetylcholine or 5-HT (Por et al., 1982; Chaouloff, 1989; Fordyce and Farrar, 1991; Rasmuson et al., 1998), or increase of synaptic plasticity (Vivar et al., 2013; Nelson and Alkon, 2015), but there are also differences, which could explain the different pattern of activation of neurogenesis.

One major difference between exercise and environmental enrichment is for instance that the former, but not the latter, increases the levels of BDNF, which is directly involved in neuron survival and plasticity but only indirectly in progenitor cell proliferation (Bechara et al., 2014). These data were obtained in the absence of exercise. Thus, other components may be involved in the pro-survival effect of environmental enrichment, such as the cyclic adenosine monophosphate response element-binding protein (CREB), or the chemokine Cxcl12 (Zhang et al., 2016). No pro-neurogenic effect of environmental enrichment was observed in the SVZ (Zhang et al., 2016).

Interestingly, however, a more recent report indicates that an enriched environment induces a significant increase of the number of dentate gyrus stem cells (GFAP^+^Sox2^+^) as well as of progenitor cells, and an improved spatial memory; this effect is more pronounced if hypoxia precedes exposure to an enriched environment (Salmaso et al., 2012). We should however consider that when physical activity is mixed to environmental enrichment, as occurs in the report by Salmaso et al. (2012), then it becomes difficult to distinguish between the effects of the two stimuli. A further evidence excluding any pro-proliferative direct effect by environmental enrichment shows that its effects on hippocampal-dependent memory are independent on adult neurogenesis (Meshi et al., 2006). Instead, spatial training has been shown to cause an increase of the generation of BrdU-positive cells, which can be interpreted in terms of increased proliferation of progenitor cells (Gould et al., 1999) as well as of increased survival of new neurons (Ambrogini et al., 2000). However, a critical time-window has been identified for spatial training, since if this occurs 1 week after birth, it induces increase of neuron survival, while if occurring in the second week after birth, it decreases survival, probably as a consequence of a competitive integration of one-week old neurons (Döbrössy et al., 2003; Ambrogini et al., 2004; Epp et al., 2007, 2013). It is not clear, however, whether spatial training is able to induce the activation of stem cells, in addition to progenitor cells (Table 1).

### Nutrients as Activators of Stem Cells in Adult and Aged Dentate Gyrus and SVZ

As for nutrients, they can have a powerful effect on neurogenesis: caloric/dietary restriction, omega-3 fatty acids (abundant in fish), polyphenols (present in extra virgin olive oil), including flavonoids (contained in wine), all increase neurogenesis (reviewed by Stangl and Thuret, 2009; Dias et al., 2012; Phillips, 2017; Sarubbo et al., 2018). Concerning their ability to stimulate stem cells, it has been shown that, for instance, in the mouse model of Down syndrome and Alzheimer disease Ts65Dn, the natural flavonoid luteolin rescued the decreased production of dentate gyrus stem and progenitor cells and also improved spatial memory as well as novel object recognition ability (Zhou et al., 2019).

Moreover, in a recent study of the effect on dentate gyrus cells of hydroxytyrosol (HTyr), a natural anti-oxidant phenolic compound present in extra virgin olive oil, we observed that HTyr in adult mice increases the number of new neurons by enhancing their survival without effect on the proliferation of stem and progenitor cells (D’Andrea et al., 2020; Table 1). However, in aged mice as well as in the Btg1 knockout neural aging model, HTyr increases not only the survival of new neurons and improves their integration into memory circuits, but also strongly increases the proliferation of stem and progenitor cells and reduces aging markers such as lipofuscin and Iba-1 (D’Andrea et al., 2020; Tables 1, 3). Thus, HTyr is able to counteract the effect of aging on neurogenesis.

**TABLE 3:** Proliferative activation of stem and progenitor cells by neurogenic stimuli when genes controlling stem cells in dentate gyrus or SVZ are deleted.

Gene mutation (dentate gyrus)	Neurogenic stimulus	Activation of stem cells (type-1; BrdU^+^GFAP^+^nestin^+^Sox2^+^)	Activation of progenitor cells (type-2-3)	Cognitive effects	References
Btg1 KO	Nutrient: Hydroxytyrosol	YES	YES	–	D’Andrea et al., 2020
	Running	YES	YES	Rescue of defective contextual discrimination	Farioli-Vecchioli et al., 2014
	Fluoxetine (in adult and aged mice)	YES	YES (also increased number of neurons)	–	Micheli et al., 2018a
p16 KO (in aged mice)	Running	YES	YES	–	Micheli et al., 2019
Notch1 KO	Running	NO	YES	–	Ables et al., 2010
p57 KO	Running	NO	–	–	Furutachi et al., 2013
LCN2 KO	Running	NO (28 days running)	YES	Reduced anxiety and improved contextual discrimination	Ferreira et al., 2019
5-HT3 receptor KO	Running	NO	NO	No antidepressant effect	Kondo et al., 2015
GABA_*A*_ receptor activation by DBI KO	Running and environmental enrichment	NO	NO	–	Dumitru et al., 2017
Tryptophan hydroxylase 2 KO	Running	NO	NO	–	Klempin et al., 2013
5-HT1 receptor KO	Fluoxetine	–	NO		Santarelli et al., 2003

**Gene mutation (SVZ)**	**Neurogenic stimulus**	**Activation of SVZ stem cells (Ki67/BrdU^+^ GFAP or nestin/sox2^+^)**	**Activation of SVZ neuroblasts/neurons (Ki67/Brdu^+^ and/or DCX^+^ or NeuN^+^)**	**Cognitive effects**	**References**

Btg1 KO (in adult as well as aged mice)	Running	YES	YES	Rescue of neurons recruited to olfactory circuits	Mastrorilli et al., 2017
p21 KO (in adult)	Running	YES	YES	Improvement of olfactory threshold	Nicolis di Robilant et al., 2019

Similarly, another natural phenolic compound, caffeoylquinic acid, is able to improve spatial memory and induce an increase of the number of dentate gyrus stem cells (BrdU^+^GFAP^+^ cells) as well as of neurons (BrdU^+^NeuN^+^), in the senescence-accelerated prone eight mouse (SAMP8) (Sasaki et al., 2019; Table 1).

Also Astragaloside VI, belonging to a group of triterpene glycosides, is able to increase after ischemia the number of stem cells (BrdU^+^GFAP^+^ and BrdU^+^Sox2^+^ cells) in the dentate gyrus and SVZ, rescuing the deficit of spatial memory, with a mechanism that involves the EGF receptor (Chen et al., 2019; Tables 1, 2).

It is worth noting also that the prolonged exposure to caloric restriction in aged mice increases the number of dentate gyrus dividing cells (nestin^+^, i.e., stem and progenitor cells) but not of neuroblasts (DCX^+^) in female mice (Park et al., 2013).

Other studies showing increased SGZ neurogenesis by nutrients did not define whether these are able to induce stem cells or not: for instance, omega-3 polyunsaturated acids (n-3 PUFA) increase proliferation of progenitor cells (DCX^+^), dendritic length, and spatial memory (Cutuli et al., 2014); or the natural green tea epigallocatechin-3-gallate compound appears to increase the number of progenitor cells in the dentate gyrus (Wang et al., 2012).

Overall, rescue of neurogenesis by nutrients in conditions of decreased function, such as neurodegeneration, trauma, ischemia or aging, can lead to activation of stem cells, while in physiological conditions (e.g., HTyr treatment in adult mice) nutrients appear unable to activate stem cells. This latter is somewhat surprising, if we consider that the metabolism plays a great role in the activation of stem cells (Arnold et al., 2015), as indicated by different laboratories, showing that the activation of stem cells depends on the reduction of the oxidative state (Adusumilli et al., 2019 [Preprint]) and on the activation of the metabolism of fatty acids (Knobloch et al., 2017) as well as on the inhibition of glycolysis (Llorens-Bobadilla et al., 2015) or on low density lipoprotein (LDL) downregulation (Engel et al., 2019).

However, we can speculate that in conditions of normal metabolism, nutrients, as a whole or as specific compounds, may result unable to stably alter the homeostasis of the oxidative state or of fatty acids and/or glycolysis sufficiently to trigger activation of stem cells, while in pathological conditions of deregulation, e.g., when neuroinflammation with increased ROS takes place, the system may become less resilient against changes.

In fact, polyphenols exert multiple antioxidant effects, by directly quenching ROS (Hollman et al., 2011), and by inhibiting the enzymes that generate ROS (monoamine oxidase or xanthine oxidase; Sandoval-Acuña et al., 2014), thus linking antioxidant nutrients to the activation of stem cells. It is also worth noting that polyphenols activate SIRT1 (Howitz et al., 2003) which in turn inhibits NF-kB (Chen et al., 2005), whose downregulation reduces the inflammatory state (Xie et al., 2013) and favors the self-renewal of stem cells (Soria-Valles et al., 2015).

### Electromagnetic Fields as Activators of Dentate Gyrus Stem Cells

Interestingly, also low frequency electromagnetic fields have been shown to improve spatial learning and memory and enhance hippocampal neurogenesis, including the proliferation of neural stem cells *in vitro*, possibly through activation of the CREB pathway (Leone et al., 2014; Table 1). Consistently, low frequency electromagnetic fields have been found to induce an increase of proliferation of hippocampal progenitor cells cultured *in vitro*, from either normal or ischemic brains. It appears that the activation of the AKT pathway is required for the proliferation increase in cultures of progenitor cells from ischemic brains (Cheng et al., 2015). The activation of the AKT pathway, which plays a key role for cell survival signaling, is common to other neurogenic stimuli, such as running (Chen and Russo-Neustadt, 2009) and nutrients (e.g., hydroxytyrosol; Fu and Hu, 2016) indicating the existence of shared mechanism underlying the increase of neurogenesis.

### High Intensity Activators of Dentate Gyrus and SVZ Stem Cells

It has moreover been shown that some models of pathological conditions or therapeutic treatments act as strong neurogenic stimuli, which are able to activate stem cells also in adult mice, where “normal” neurogenic stimuli such as running are ineffective. These stimuli include electroconvulsive shock, which leads to massive cell depolarization (Segi-Nishida et al., 2008), traumatic brain injury (in the dentate gyrus, Gao et al., 2009, and in the early postnatal SVZ, Goodus et al., 2015), kainic acid (Hüttmann et al., 2003), and tetanus toxin (Jiruska et al., 2013) injections, which induce seizures, or also clozapine-*N*-oxide-mediated activation of the Gq protein in stem cells (Dong et al., 2019). Also focal ischemia typically induces an increase of neurogenesis in the dentate gyrus (Kee et al., 2001) as well as in the SVZ (Thored et al., 2006).

In the case of the traumatic brain injury, this stimulus has been observed to alter SVZ proliferation in a variable fashion across species and experiments (Chang et al., 2016). Conversely, the strong enhancement of cell survival by Bax deletion, despite that was driven by nestin promoter in stem/progenitor cells, did not stimulate stem cell proliferation, thus indicating that the Bax-dependent increase of survival is in itself not sufficient to provoke stem cell activation (Sahay et al., 2011a). Of note, brain trauma is able to activate SVZ stem and progenitor cell proliferation, with an increase of the neuroblasts migrating outside the SVZ to the injury site. It appears, however that this did not result in significant neuron replacement, indicating that new strategies are needed to improve the regenerative response (Goodus et al., 2015). Furthermore, it is remarkable that traumatic brain injury can stimulate the generation of stem cells also in the dentate gyrus, while no effect is observed on progenitor cells. As for the underlying mechanism, the authors speculate that the long apical processes of quiescent stem cells extending in the molecular layer of the dentate gyrus may be sensitive to diffusible factors in the microenvironment (Gao et al., 2009). Recently, a very interesting study unveiled a mechanism of activation of stem cells in the SVZ by focal ischemia, showing that dormant/quiescent stem cells progress after ischemia to a primed state, triggered by interferon gamma signaling, after which they are activated; activation occurs following a decrease of Notch and BMP signaling (Llorens-Bobadilla et al., 2015). This report, thus, explains how a strong neurogenic stimulus can indeed activate a population of stem cells physiologically quiescent (see Table 4).

**TABLE 4:** High intensity activators of dentate gyrus and SVZ stem cells.

System	Neurogenic stimulus	Activation of stem cells (type-1; BrdU^+^GFAP^+^ nestin^+^Sox2^+^)	Activation of progenitor cells (type-2-3) or neuroblasts	Cognitive effects	References
Adult	Electroconvulsive shock	YES	YES	–	Segi-Nishida et al., 2008
	Traumatic brain injury	YES (dentate gyrus)	NO (dentate gyrus)	–	Gao et al., 2009;
	Traumatic brain injury	YES (SVZ)	YES (SVZ; increased migration of neuroblasts out of SVZ)		Goodus et al., 2015
	Focal brain ischemia	YES (SVZ)	YES (SVZ)	–	Llorens-Bobadilla et al., 2015
	Kainic acid	YES	YES (faintly EGFP-positive)	–	Hüttmann et al., 2003
	Tetanus toxin	YES	YES	–	Jiruska et al., 2013
	Activation of stem cells (by CNO-mediated activation of Gq protein hM3Dq)	YES	YES	–	Dong et al., 2019
	Enhancement of neuron survival by Bax deletion	NO	YES	Increase of adult hippocampal neurogenesis improves discrimination between similar contexts	Sahay et al., 2011a

Moreover, the involvement has been recently shown of Ascl1 (Mash1) in kainic acid stimulation (Andersen et al., 2014). This report shows that Ascl1 is not required for the maintenance of radial glia stem cells, but specifically for their activation. In fact, when a stimulus such as kainic acid (but not running) arrives to the stem cell, Ascl1 expression is induced, and this increase is required for the cell to exit from quiescence. The fact that Ascl1 binds to and activates the promoter of cyclin D2 suggests that Ascl1 triggers the cell cycle activation directly (Andersen et al., 2014).

As a whole these reports suggest that high intensity stimuli exert an integrated control of stem cell activation, active on both quiescence-maintaining and cell cycle genes.

## Interaction Between Genes Controlling Stem Cell Activation in the Dentate Gyrus or SVZ and Neurogenic Stimuli

How is a neurogenic stimulus such as physical exercise interacting with the network of stem cell quiescence-maintaining genes in the dentate gyrus? Running, when the cell cycle inhibitory genes Btg1 or p16Ink4a are deleted, does activate stem cells above the level attained by knockout sedentary mice (Farioli-Vecchioli et al., 2014; Micheli et al., 2019), but is ineffective when p57Kip2 (Furutachi et al., 2013), or Notch1 (Ables et al., 2010) are ablated; or, in the case of lipocalin 2 (LCN2) knockout, a decrease of stem cells, after 28 days of running, versus sedentary mice is observed (Ferreira et al., 2019; Table 3). This clearly indicates that Btg1 and p16Ink4a prevent activation of stem cells by the wide-range neurogenic stimulus of running, given that their ablation enables stem cells to become responsive to that stimulus. Additionally, in the case of Btg1 and p57Kip2 knockouts, radial neural stem cells undergo a transient expansion (in early postnatal mice for Btg1 knockout and immediately after ablation for p57Kip2 knockout), indicating that these genes maintain stem cells quiescent in basal conditions. Furthermore, as mentioned above, Btg1, Notch1, and p57Kip2 knockouts show a decrease of the dentate gyrus stem cell number in sedentary conditions, either soon after ablation in conditional knockout (Notch1; Ables et al., 2010), or sometime after birth in constitutive knockout (Btg1; Farioli-Vecchioli et al., 2012), or a long time after deletion in p57Kip2 conditional knockout (24 months after; Furutachi et al., 2013). This suggests a depletion of the stem cell pool after prolonged activation, although to different extents. This possibility, however, is contradicted by the prompt reactivation of stem cells elicited by running, at least in Btg1 and in p16Ink4a knockouts, in this latter case in aged mice with physiologically reduced neurogenesis. It is also worth noting that reactivation by running in p16ink4a and Btg1 knockouts appears to be long lasting after the end of the stimulus (Farioli-Vecchioli et al., 2014; Micheli et al., 2019).

In the SVZ, as mentioned above, running is able to activate stem cells in aged but not in adult mice (Brown et al., 2003; Blackmore et al., 2009; Mastrorilli et al., 2017; Table 2). Moreover, the deletion of p21Cip1 or Btg1 enables running to activate adult as well as aged SVZ stem and progenitor cells, with improvement of olfactory circuits and threshold (Mastrorilli et al., 2017; Nicolis di Robilant et al., 2019; Table 3).

Thus, dentate gyrus and SVZ stem cells are normally not activated by running, but become activatable after deletion of specific genes negatively controlling the cell cycle, such as p16Ink4a, Btg1 and p21Cip1. A quiescence-maintaining action on progenitor cells is exerted also by GABA_*A*_ receptors, whose activation following DBI knockout prevents the proliferative effect on progenitor cells by running (Dumitru et al., 2017; see section “Physical Exercise in the Dentate Gyrus and SVZ” and Table 3). Conversely, 5-HT3 receptors exert a proliferative stimulus on SGZ cells, and their deletion impairs the proliferative activation of progenitor cells by running (Kondo et al., 2015; see section “Physical Exercise in the Dentate Gyrus and SVZ” and Table 3).

Concerning the interaction of fluoxetine with the genes controlling stem cell quiescence, when Btg1 is deleted, fluoxetine acquires the ability to activate dentate gyrus stem cells in adult as well as in aged mice (15-month-old; Micheli et al., 2018a), indicating that Btg1 restrains this neurogenic stimulus from being effective on stem cells (Table 3). Moreover, as mentioned above, fluoxetine treatment inhibits the expression of p21Cip1 in the dentate gyrus, and the deletion of p21Cip1 triggers the proliferation of stem and progenitor cells (Pechnick et al., 2011). The fluoxetine-dependent pro-neurogenic stimulus is controlled by 5-HT1 receptor, as its knockout inactivates the proliferative stimulus of fluoxetine on progenitor cells of the dentate gyrus (Santarelli et al., 2003; Table 3).

Another neurogenic stimulus, HTyr, has been shown to be able to stimulate dentate gyrus stem cells in aged and in the Btg1 knockout mice (see above; D’Andrea et al., 2020; Table 3).

All this supports the concept of a gene network preventing stem cell activation by neurogenic stimuli, not only in the dentate gyrus but also in the SVZ. The idea conveyed by these data is that when cell cycle regulatory genes (e.g., p16Ink4a) are deleted, the pro-proliferative pathways prevail, and stimuli normally unable to activate stem cells, such as running or fluoxetine, become effective. This would perhaps account for the lack of responsiveness of dentate gyrus stem cells to running after deletion of Notch, which does not directly impact on the cell cycle (Ables et al., 2010).

## Conclusion

It appears that stem cells can be reactivated in the dentate gyrus or SVZ by appropriate stimuli, under conditions, in apparent paradox, favoring their depletion, such as aging or depression, i.e., when neurogenesis is lower, and/or after deletion of quiescence-maintaining genes. For instance, HTyr or running activate stem cells in aged dentate gyrus or aged SVZ, respectively (Blackmore et al., 2009; D’Andrea et al., 2020). Actually, the preservation of the quiescent state of stem cells appears less effective when the system is aging or carrying mutations, i.e., when a neurogenic stimulus can activate also stem cells.

A deforestation theory has been proposed, whereby the age-dependent decline in neurogenesis can be ascribed to a diminution of the pool of stem cells that are being activated, mainly for incapability to divide (Lugert et al., 2010) or for conversion to astrocytes (Encinas et al., 2011; Encinas and Sierra, 2012). The number of proliferating stem cells is certainly reduced during aging (e.g., see Lugert et al., 2010 or Micheli et al., 2018a). Nevertheless, stem cells can be reactivated in large numbers following a neurogenic stimulus, as mentioned above; other examples of reactivation during aging are by kainic acid (Lugert et al., 2010), or by running in p16Ink4a-*null* dentate gyrus and in p21Cip1-*null* SVZ (Micheli et al., 2019; Nicolis di Robilant et al., 2019); furthermore, a short VEGF preconditioning allows the rescue of the age-induced quiescence of neural stem cells of the dentate gyrus without causing depletion (Licht et al., 2016).

This would suggest that during aging there is, rather than an incapability of stem cells to expand (Encinas and Sierra, 2012; Lugert et al., 2010), an increased need and/or presence of conditions safeguarding from stimuli, which are enforced by genes such as the cell cycle inhibitors p16Ink4a or p21Cip1. In fact, p16Ink4a expression increases during aging (Molofsky et al., 2006), plausibly in response to a reduced ability of the system to maintain the stem cell pool in homeostasis. A functional reason for this attempt to preserve quiescence may also be the maintenance of the existing hippocampal memory circuitry, favoring the current situation versus renewal.

It appears, moreover, that strong conditions in favor of stem cell activation (e.g., p57Kip2 long-term deletion or kainic acid treatment; Furutachi et al., 2013; Sierra et al., 2015), do not achieve a complete pool depletion, suggesting that stem cells are resilient to exhaustion. It should be also noted that neural stem cells in tumors survive extensive irradiation and, given the appropriate stimulus, they will restart dividing even after several years of quiescence (Aguirre-Ghiso, 2007). Thus, a possibility which should be thoroughly tested is whether stem cells of the neurogenic niche may, even after a great number of rounds of proliferation, resume dividing following a period of quiescence and attain expansion from a few remaining cells.

The resilience of the neural stem cell pool is an issue of therapeutic relevance, not only for aging, but also in light of the correlation observed between neurogenesis and depression, and of the possibility to choose an appropriate neurogenic stimulus (e.g., diet or physical exercise).

More generally, the data available show that the activation of stem cells can be elicited by normal stimuli after deletion of cell cycle inhibitory genes – thus allowing proliferative signals to prevail (e.g., 5-HT) – or directly by strong stimuli. These latter, apparently, are able to bypass the inhibition of the cell cycle, as shown for kainic acid treatment, which, in SGZ and SVZ stem cells activates Ascl1 that in turn activates directly cyclin D2 (Andersen et al., 2014). Another example of a mechanism of direct activation of stem cells is that provided by Llorens-Bobadilla et al. (2015), whereby interferon gamma signaling is induced in the SVZ after ischemia. Moreover, it is also plausible that, as previously suggested, different sets of stem cells respond to different stimuli (Lugert et al., 2010), thus providing a different response according to the intensity and quality of the stimulus. We cannot exclude that cell cycle inhibitory genes may maintain the quiescence of specific subpopulations of stem cells; gene deletion driven by specific markers will be necessary to assess this possibility.

## Author Contributions

FT had the conceptual idea, carried out the literature search and wrote the manuscript. MC, GD’A, and LM contributed to the conceptual idea, gave suggestions and refined the manuscript.

## Conflict of Interest

The authors declare that the research was conducted in the absence of any commercial or financial relationships that could be construed as a potential conflict of interest.
